# Histological Chorioamnionitis Induces Differential Gene Expression in Human Cord Blood Mononuclear Leukocytes from Term Neonates

**DOI:** 10.1038/s41598-019-42205-x

**Published:** 2019-04-10

**Authors:** Suhita Gayen nee’ Betal, Swati Murthy, Michael Favara, Gina Fong, Joanna S. Y. Chan, Sankar Addya, Thomas H. Shaffer, Jay Greenspan, Vineet Bhandari, Irfan Rahman, Zubair H. Aghai

**Affiliations:** 1 Neonatology, Thomas Jefferson University/Nemours, Philadelphia, PA USA; 2 Department of Pathology, Thomas Jefferson University, Philadelphia, PA USA; 3 Cancer Genomics and Bioinformatics Laboratory, Sidney Kimmel Cancer Center, Thomas Jefferson University, Philadelphia, PA USA; 4 Section of Neonatology, Department of Pediatrics, St. Christopher’s Hospital for Children, Drexel University College of Medicine, Philadelphia, PA USA; 5 Department of Environmental Medicine, University of Rochester Medical Center, Rochester, NY United States

## Abstract

Histological chorioamnionitis (HCA) is an infection of fetal membranes and complicates 5.2% to 28.5% of all live births. HCA is associated with increased mortality and morbidity in both premature and term neonates. Exposure to HCA may have long-term consequences, including an increased risk for allergic disorders and asthma later in childhood, the mechanism(s) of which are still not yet well understood. The objective of this study was to determine the mRNA transcriptome of cord blood mononuclear leukocytes from term neonates to identify key genes and pathways involved in HCA. We found 366 differentially expressed probe IDs with exposure to HCA (198 upregulated, 168 downregulated). These transcriptomes included novel genes and pathways associated with exposure to HCA. The differential gene expression included key genes regulating inflammatory, immune, respiratory and neurological pathways, which may contribute to disorders in those pathways in neonates exposed to HCA. Our data may lead to understanding of the role of key genes and pathways identified on the long-term sequelae related to exposure to HCA, as well as to identifying potential markers and therapies to prevent HCA-associated complications.

## Introduction

Chorioamnionitis (CA) is an infection of the fetal membranes and placenta that complicates 5.2% of all live births^1^. The prevalence of histological CA (HCA) in term neonates with spontaneous labor is 23.6–28.7%^2,3^. Approximately 500,000 to one million infants in the United States are born each year to mothers diagnosed with HCA. Exposure to HCA may have long-term consequences, including an increased risk for allergic disorders and asthma later in childhood^4–6^. Additionally, studies have demonstrated that exposure to HCA can also lead to developmental delay and cerebral palsy in preterm and term infants^7–9^. Asthma and other allergic chronic diseases during childhood affect greater than 7 million children in the United States^10^. Although HCA is associated with the development of asthma, allergic disorders, and neurodevelopmental impairment, the exact mechanism for these complications is unknown. Exposure to infection during late fetal life is likely to incite epigenetic changes, which may modulate the immune and neurological systems and increase the risk for development of neurological and allergic disorders later in life.

Gene expression studies of cells and tissues have become a major tool for discovery in the pathogenesis of various diseases. Global gene expression by transcriptomic analysis can uncover gene signatures and help delineate molecular pathways involved in the development of asthma, allergy, and neurodevelopmental impairment in neonates born to mothers with HCA. Previous studies have reported differential gene expression patterns associated with preterm labor and lipopolysaccharide (LPS) stimulation of cord blood leukocytes^11,12^. More recently, differential gene expression in whole blood from preterm neonates exposed to HCA has been published^13^. However, fetal inflammatory and immune response to microbial infection differ between preterm (born before 37 weeks gestation) and term infants^14–16^. The effects of HCA on gene expression profile on cord blood mononuclear leukocytes in human term neonates have not been characterized. Our objective was to determine the mRNA transcriptome of cord blood mononuclear leukocytes from term neonates and identify key genes and pathways involved in HCA.

## Results

Ten term infants were enrolled in the study. Five infants had histological chorioamnionitis (HCA group), and five infants with no evidence of histological chorioamnionitis on placental histopathology served as the control group.

### Differential Gene Expression

Comparison of the HCA group array data with the control group using transcriptome array console software revealed that 366 probe IDs were differentially expressed with a fold change ≥ 1.5 (p < 0.05). Of these, 198 probe IDs were significantly up-regulated (Supplementary Table 1), 60 with annotated genes and 138 non-annotated genes. One hundred and sixty-eight probe IDs were significantly down-regulated (Supplementary Table 2), of which 105 had annotation with gene symbols and 63 were not annotated. The top 10 up- and down-regulated genes based on the fold change are reported in Table 1. The top 10 up-regulated genes included chemokine (C-C motif) receptor-2 (CCR2), a pro-inflammatory chemokine important in inflammatory diseases including asthma^17,18^. The top down-regulated genes included two important antimicrobial proteins, lactoferrin (LTF) and cathelicidin antimicrobial peptide (CAMP).

**Table 1:** Top 10 differentially up- or down-regulated genes after exposure to HCA.

ProbeID	Gene Symbol	HCA Group Average Expression	Control Group Average Expression	Fold Change	Up/Down	P-value
TC17001604.hg.1	**LRRC37A4P**	1389.16	344.89	**4.01**	**Up**	0.0245
TC04002938.hg.1	**LOC285505**	364.56	136.24	**2.68**		0.012
TC07000288.hg.1	**LINC01061**	1652	643.59	**2.58**		0.0117
TC03000255.hg.1	**CCR2**	765.36	304.44	**2.51**		0.0417
TC04001682.hg.1	**FAM198B**	643.59	256	**2.5**		0.0167
TC17000604.hg.1	**LRRC37A2**	13216.02	5556.65	**2.37**		0.0312
TC07001299.hg.1	**TRGV4**	140.07	60.13	**2.32**		0.0002
TC07001300.hg.1	**TRGV3**	179.77	79.89	**2.24**		4.50xE-05
TC04002939.hg.1	**TMEM144**	240.52	112.99	**2.12**		0.0173
TC17001603.hg.1	**LRRC37A4P; LRRC37A2**	20594.91	9946.68	**2.08**		0.0478
TC01003638.hg.1	**PTGS2**	252.48	9089.59	**−35.91**	**Down**	0.0205
TC04002953.hg.1	**AREG**	313	1884.54	**−6.03**		0.001
TC01001749.hg.1	**G0S2**	2352.53	13034.07	**−5.54**		0.008
TC05001854.hg.1	**HBEGF**	385.34	2005.85	**−5.18**		0.0022
TC19001593.hg.1	**PLAUR**	1448.15	7082.29	**−4.89**		0.0138
TC21001069.hg.1	**SAMSN1**	3956.48	17438.64	**−4.42**		0.0257
TC12001216.hg.1	**OLR1**	178.53	765.36	**−4.27**		0.0365
TC03000276.hg.1	**CAMP**	657.11	2683.69	**−4.09**		0.0011
TC09000508.hg.1	**NR4A3**	143.01	580.04	**−4.07**		0.0128
TC03001357.hg.1	**LTF**	2683.69	9345.14	**−3.49**		0.0208

### Differential Gene Expressions Related to Immune Response

Thirty genes related to immune response and important for regulation of inflammatory pathways were identified (Table 2). Twelve genes were up-regulated and 18 genes were down-regulated. The up-regulated genes have been previously associated with asthma; hyper-reactivity and allergy (CCR2, HRH2, PTEN); lung development and apoptosis (CHRNA7, RTKN2); and immune function (GIMAP4, GIMAP7, KLRC3 and CD99) (Table 3). Similarly, the down-regulated genes have roles in asthma and airway modeling (PTGS2, AREG, HBEGF, PLAUR, CAMP, LTF, FCAR, PDE4B, ZNF331 and IRAK2) and immune function (NR4A3, NFIL3, ADGRE3, TARM1 and ZC3H12A). The down-regulated gene CXCL1 has a role in lung development and BPD (Table 4).

**Table 2:** Differential expression of genes related to immune response after exposure to HCA.

Gene Symbol	Up/Down	Fold Change	P-value	HCA Group Average Expression	Control Group Average Expression
**CCR2**	**Up-regulated Genes**	**2.51**	0.0417	765.36	304.44
**TRGV4**		**2.32**	0.0002	140.07	60.13
**GIMAP4**		**2.29**	0.0118	52498.92	23010.42
**TRGV3**		**2.24**	0.000045	179.77	79.89
**KLRC3**		**1.8**	0.0433	1458.23	809.00
**HRH2**		**1.74**	0.0233	1734.13	1002.93
**CHRNA7**		**1.61**	0.0201	33.36	20.68
**GIMAP7**		**1.61**	0.0086	4482.23	2778.33
**RTKN2**		**1.59**	0.0294	153.28	96.34
**CD99**		**1.56**	0.0121	2646.74	1698.45
**PTEN**		**1.55**	0.0275	694.58	448.82
**GIMAP1-GIMAP5; GIMAP5; GIMAP1**		**1.53**	0.0155	989.12	648.07
**PTGS2**	**Down-regulated Genes**	**−35.91**	0.0205	252.48	9089.59
**AREG**		**−6.03**	0.001	313.00	1884.54
**HBEGF**		**−5.18**	0.0022	385.34	2005.85
**PLAUR**		**−4.89**	0.0138	1448.15	7082.29
**CAMP**		**−4.09**	0.0011	657.11	2683.69
**NR4A3**		**−4.07**	0.0128	143.01	580.04
**LTF**		**−3.49**	0.0208	2683.69	9345.14
**NFIL3**		**−2.77**	0.0262	1192.69	3304.00
**FCAR**		**−2.35**	0.0023	2256.70	5330.30
**PDE4B**		**−2.33**	0.0125	280.14	652.58
**ADGRE3**		**−2.3**	0.042	680.29	1573.76
**CXCL1**		**−2.05**	0.0404	112.21	229.13
**TARM1**		**−2.02**	0.0059	116.16	235.57
**SNAI1**		**−1.93**	0.0083	47.18	91.14
**ZNF331**		**−1.77**	0.0308	288.01	512.00
**ZC3H12A**		**−1.76**	0.0422	256.00	451.94
**IRAK2**		**−1.74**	0.048	164.28	284.05
**NFKBIE**		**−1.59**	0.0351	413.00	657.11

**Table 3:** Up-regulated genes after exposure to HCA and their functions.

Gene Symbol	Gene name	Function
**CCR2**	C-C Motif Chemokine Receptor 2, Receptor for monocyte chemoattractant protein-1	Role in asthma, inflammation
**GIMAP4**	GTPase, Immunity-Associated Protein family member 4	T- and B-cell development and survival, T-cell apoptosis
**KLRC3**	Killer Cell Lectin-Like Receptor Subfamily C, Member 3	Natural Killer receptor gene, role in cell proliferation
**TRGV3**	T Cell Receptor Gamma Variable 3	Peptide antigen binding, immune response
**HRH2**	Histamine Receptor H2	Histamine receptor gene, role in hypersensitiviy
**CHRNA7**	Cholinergic Receptor Nicotinic Alpha 7 Subunit	Alter airway morphometry and lung function
**GIMAP7**	GTPase, Immune-Associated Nucleotide-Binding Protein 7	Regulators of lymphocyte survival and homeostasis (Schwefel D 2013)
**RTKN2**	Rhotekin 2	Role in apoptosis
**CD99**	Cluster of differentiation 99, Single-chain type-1 glycoprotein	Role in cell adhesion, migration, death, differentiation and diapedesis, and it influences processes associated with inflammation, immune responses (pasello M 2018)
**VNN2**	Vanin 2	Hematopoietic cell trafficking, Role in oxidative-stress
**PTEN**	Phosphatase And Tensin Homolog	Role in asthma, hyper-reactivity

**Table 4:** Down-regulated genes after exposure to HCA and their functions.

Gene symbol	Gene name	Function
**PTGS2**	Prostaglandin-Endoperoxide Synthase 2	Role in asthma and lung inflammation, hyperoxia induced lung injury
**GOS2**	G0/G1 Switch 2	Regulation of lipid metabolism
**AREG**	Amphiregulin, Colorectum Cell-Derived Growth Factor	Role in asthma, airway modelling, BPD
**HBEGF**	Heparin-Binding Epidermal Growth Factor	Role in asthma, airway modelling
**PLAUR**	Plasminogen Activator, Urokinase Receptor	Role in asthma, COPD
**CAMP**	Cathelicidin Antimicrobial Peptide	Role in asthma, lung infection
**NR4A3**	Nuclear Receptor Subfamily 4 Group A Member 3	Regulates neutrolphils number and survival, Treg cell development through activation of Foxp3
**LTF**	Lactotransferrin	Role in asthma, BPA VAP
**FCAR**	Fc Fragment Of Immunoglobulin Alpha Receptor	SNP associated with allergic asthma (Jasek M 2004)
**NFIL3**	Nuclear Factor, Interleukin 3 Regulated	Critical regulator for IgE production and airway hyper-responsiveness.
**PDE4B**	Phosphodiesterase 4B	Role in asthma, inhibitors use as treatment
**ADGRE3**	Adhesion G Protein-Coupled Receptor E3	Modulator of immune cell funtion
**CXCL1**	C-X-C Motif Chemokine Ligand 1	Role in BPD, lung alveolization
**TARM1**	T Cell-Interacting, Activating Receptor On Myeloid Cells 1	Upregulated by LPS, recruitment to site of inflammation
**SNAI1**	Snail Family Transcriptional Repressor 1	Β-catenin target gene, lung fibrosis
**ZNF331**	Zinc Finger Protein 331	Pathogenesis of asthma
**ZC3H12A**	Zinc Finger CCCH-Type Containing 12A	Regulates the development and function of IL-5-producing T_H_2 cells through the Notch/Gata3 pathway
**IRAK2**	Interleukin 1 Receptor Associated Kinase 2	IRAK2 Attenuate the Proinflammatory Effects of IL-33 in Asthma-like Mouse Models.

### Ingenuity Pathway Analysis

Pathway analysis was performed using Ingenuity Pathway Analysis (IPA) software (QIAGEN Inc., https://www.qiagenbioinformatics.com/products/ingenuitypathway-analysis) by loading 366 probe sets that were differentially expressed with HCA^19^. Seventy-three functions were modified by differential gene expression with exposure to HCA. Important functions modified with the exposure to HCA are listed in Table 5. The modification of functions with HCA included genes related to immune system, inflammatory response, connective tissue disorders, neurodevelopmental disorders, hematological development and disorders, and respiratory development and disorders. Ingenuity Canonical Pathway Analysis identified 207 pathways that were modified after exposure to HCA, of which 19 pathways are known to be important in immune regulation and inflammatory responses (Table 6).

**Table 5:** Functions modified with exposure to HCA.

Modified Functions	Number of Genes Involved	Range of p-value for genes involved
Cellular Movement	46	1.26E-13-1.52E-03
Immune Cell Trafficking	38	1.26E-13-1.03E-03
Inflammatory Disease	46	7.43E-13-1.25E-03
Cell Death and Survival	60	1.5E-12-1.46E-03
Immunological Disease	47	2.74E-12-1.25E-03
Connective Tissue Disorders	38	3.3E-12-1.47E-03
Inflammatory Response	55	3.55E-11-1.38E-03
Neurological Disease	32	1.29E-10-1.38E-03
Cell-To-Cell Signaling and Interaction	40	3.45E-10-1.51E-03
Hematological System Development and Function	44	3.45E-10-1.51E-03
Cellular Growth and Proliferation	49	2.3E-07-1.46E-03
Hematopoiesis	27	4.16E-07-9.74E-04
Lymphoid Tissue Structure and Development	33	4.16E-07-9.74E-04
Cellular Function and Maintenance	42	4.95E-07-1.52E-03
Humoral Immune Response	15	5.54E-07-1.4E-03
Infectious Diseases	28	6.5E-07-1.39E-03
Respiratory Disease	19	6.5E-07-1.23E-03
Cell Cycle	26	6.84E-07-1.49E-03
Gene Expression	37	6.84E-07-1.5E-03
Free Radical Scavenging	14	7.69E-06-9.02E-06
Cell-mediated Immune Response	15	2.3E-05-5.63E-04
DNA Replication, Recombination, and Repair	11	1.06E-04-4.46E-04
Antimicrobial Response	9	2.39E-04-2.39E-04
Nervous System Development and Function	15	3.11E-04-1.19E-03
Post-Translational Modification	14	3.43E-04-3.43E-04
Hypersensitivity Response	5	3.8E-04-3.8E-04
Hematological Disease	39	4.13E-04-1.09E-03
Cellular Assembly and Organization	6	8.04E-04-8.04E-04
Psychological Disorders	3	9.59E-04-9.59E-04
Respiratory System Development and Function	2	9.59E-04-9.59E-04
Developmental Disorder	12	1.24E-03-1.24E-03
Behavior	17	1.47E-03-1.47E-03

**Table 6:** Canonical pathways in ingenuity pathway analysis associated with differentially expressed genes between control and HCA groups.

Ingenuity Canonical Pathways	−log_10_(p-value)	Molecules involved in pathways
Protein Kinase A Signaling	4.46	PTEN,PTGS2,PTPRE,DUSP6,MYH10, NFKBIE,PDE4B,ADD2,DUSP2,NFKBID
PI3K/AKT Signaling	3.32	PTEN,PTGS2,CDKN1A,NFKBIE,NFKBID
IL-17A Signaling in Airway Cells	3.12	PTEN,CXCL1,NFKBIE,NFKBID
MIF-mediated Glucocorticoid Regulation	3.09	PTGS2,NFKBIE,NFKBID
MIF Regulation of Innate Immunity	2.83	PTGS2,NFKBIE,NFKBID
iCOS-iCOSL Signaling in T Helper Cells	2.46	PTEN,HLA-DQB1,NFKBIE,NFKBID
TNFR2 Signaling	2	NFKBIE,NFKBID
IL-17 Signaling	1.99	PTGS2,CXCL1,TIMP1
4-1BB Signaling in T Lymphocytes	1.91	NFKBIE,NFKBID
IL-1 Signaling	1.9	NFKBIE,IRAK2,NFKBID
PPAR Signaling	1.9	PTGS2,NFKBIE,NFKBID
IL-17A Signaling in Fibroblasts	1.84	NFKBIE,NFKBID
B Cell Activating Factor Signaling	1.71	NFKBIE,NFKBID
IL-8 Signaling	1.7	HBEGF,PTGS2,CXCL1,IRAK2
p53 Signaling	1.68	PTEN,CDKN1A,TNFRSF10B
PI3K Signaling in B Lymphocytes	1.54	PTEN,NFKBIE,NFKBID
Glucocorticoid Receptor Signaling	1.49	CCNH,PTGS2,CDKN1A,NFKBIE,NFKBID
IL-10 Signaling	1.29	NFKBIE,NFKBID
T Helper Cell Differentiation	1.29	HLA-DQB1,IL23R

### IPA Networks

**Figure 1:**
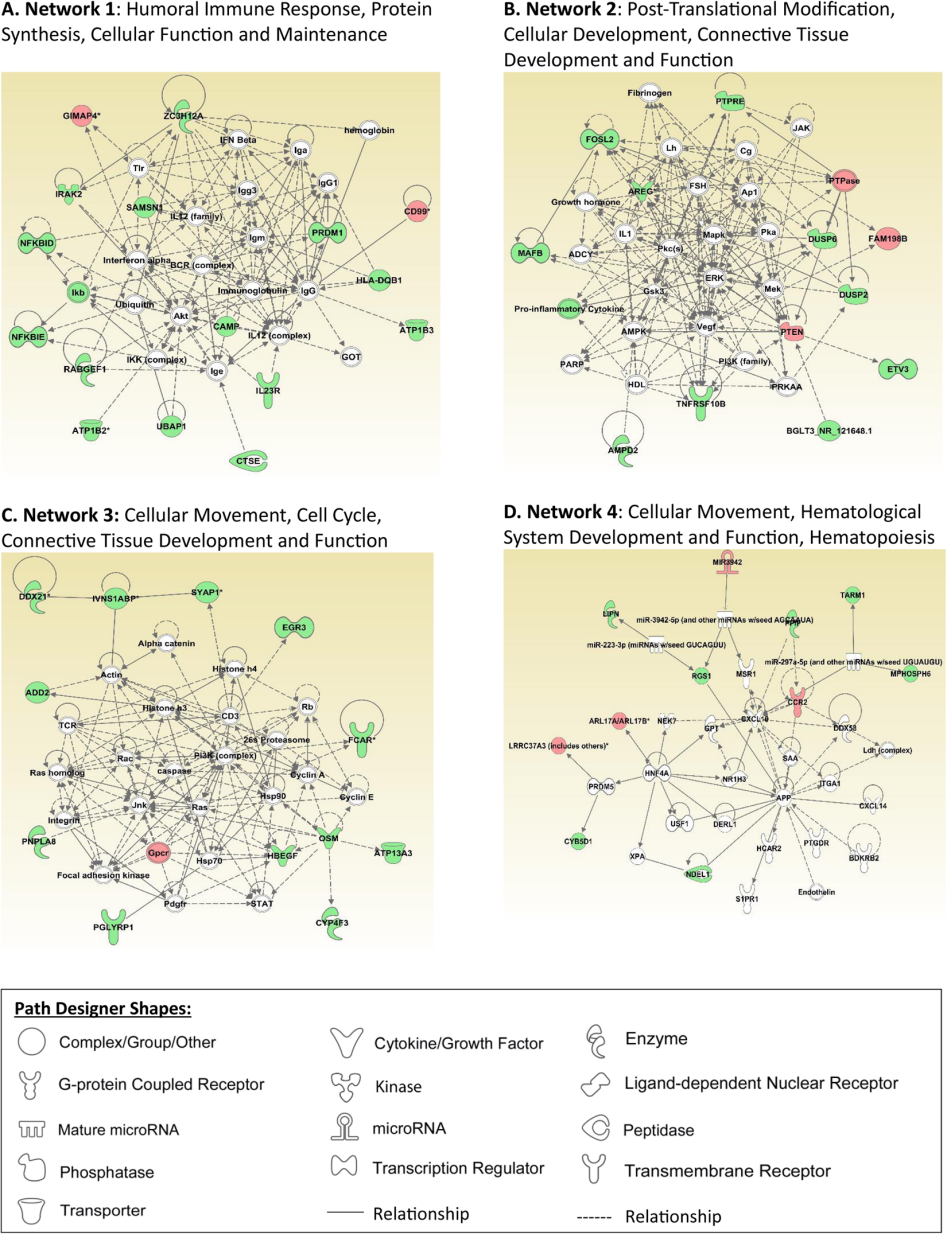
Ingenuity pathway analysis networks related to immune modulation and inflammatory response are presented in this figure (**A**–**D**). Pathway analysis was performed using Ingenuity Pathway Analysis (IPA) software (QIAGEN Inc., https://www.qiagenbioinformatics.com/products/ingenuitypathway-analysis) by loading 366 probe sets that were differentially expressed with HCA^19^.

**Figure 2:**
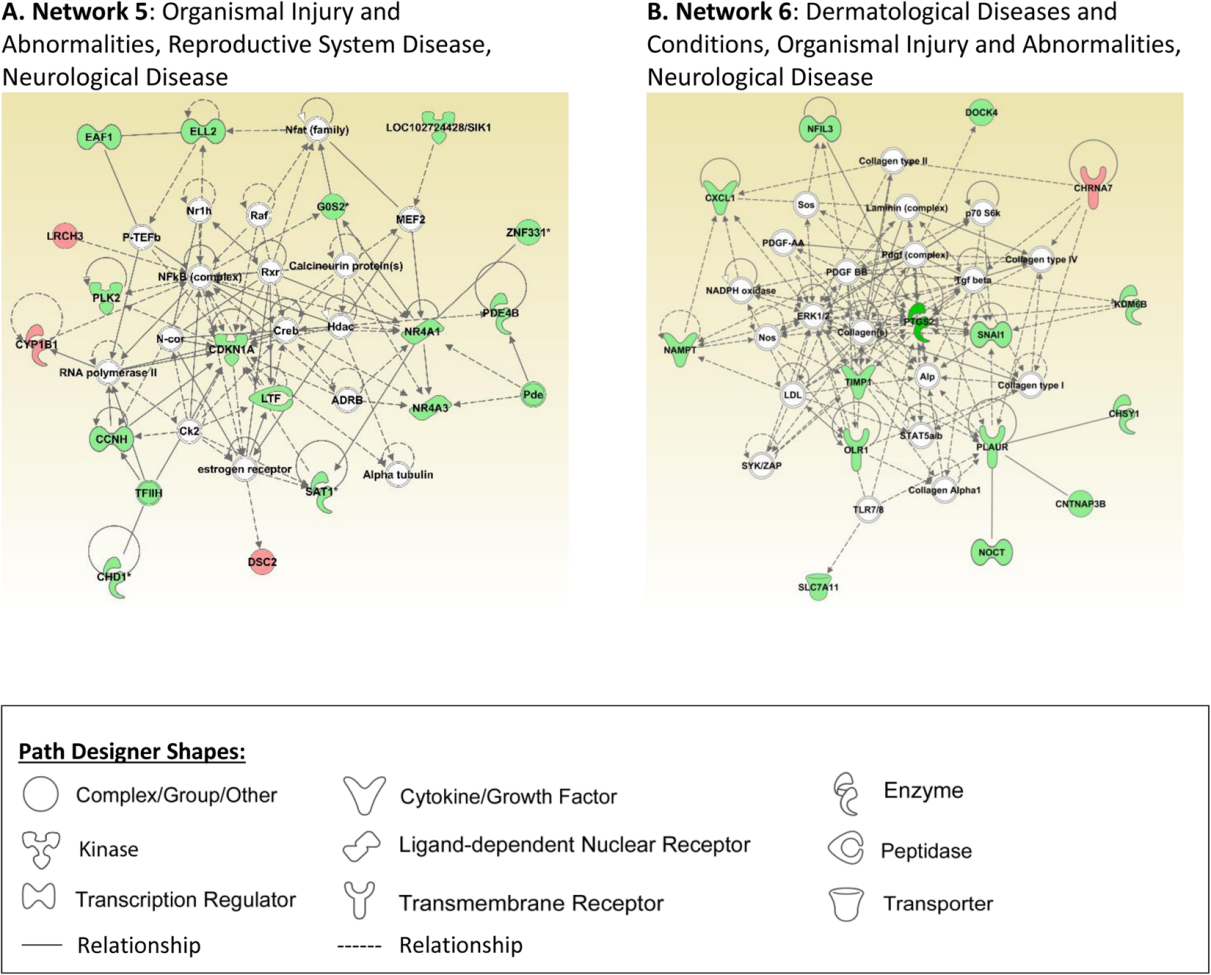
Ingenuity pathway analysis networks related to neurological disease are presented in this figure (**A,B**). Pathway analysis was performed using Ingenuity Pathway Analysis (IPA) software (QIAGEN Inc., https://www.qiagenbioinformatics.com/products/ingenuitypathway-analysis) by loading 366 probe sets that were differentially expressed with HCA^19^.

IPA picked up important networks related to immune, inflammatory, and neurological pathways. Four were closely related to immune modulation and inflammatory response and two were related to neurologic disease. Networks 1 through 4 are shown in Fig. 1. Network 1 is related to humoral immune response, protein synthesis, cellular function and maintenance. Network 2 is related to post-translational modification, cellular development, connective tissue development and function. Network 3 is related to cellular movement, cell cycle, connective tissue development, and function. Network 4 is related to cellular movement, hematological system development and function, and hematopoiesis. Networks 5 and 6 are closely related to neurological disease (Fig. 2). Network 5 involves organismal injury and abnormalities, reproductive system disease, and neurological disease. Network 6 involves dermatological diseases and conditions, organismal injury and abnormalities, as well as neurological disease.

### Quantitative Real Time PCR Verification

**Figure 3:**
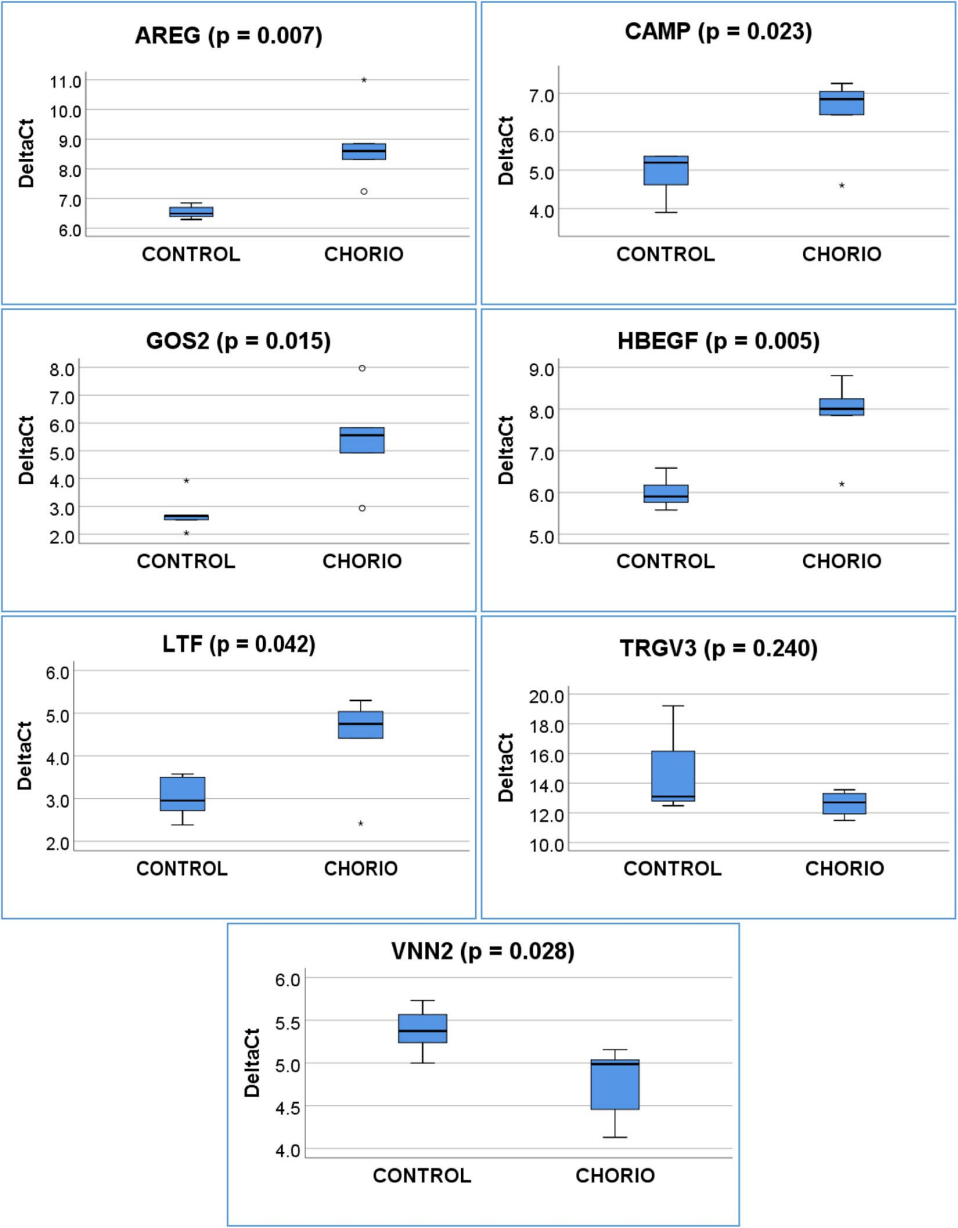
Box plot for real-time PCR data on selected genes in the two groups (Control and HCA) are presented in this figure.

To confirm gene expression results, we selected seven genes from the differentially expressed gene list that were closely related to inflammatory response and immune modulation. A quantitative Real-Time PCR was performed using total RNA to validate the microarray data from the same neonates studied by microarray analysis (Fig. 3). Of the seven selected differentially expressed genes, five were down-regulated (GOS2, AREG, HBEGF, CAMP and LTF) and two were up-regulated (VNN2 and TRGV3). Fold-changes obtained by RT-PCR were consistent with microarray results. All fold changes in RT-PCR results reached significance (p ≤ 0.05) except for TRGV3 (p = 0.24). A comparison of the microarray data with RT-PCR results is shown in Table 7.

**Table 7:** Validation of microarray data with real-time PCR.

Gene Symbol	Microarray		Real-Time PCR	
	Fold Change	p-value	Fold Change	p-value
GOS2	−3.18	0.011	−6.42	0.015
AREG	−2.54	0.006	−4.78	0.007
HBEGF	−2.15	0.008	−3.53	0.005
CAMP	−1.83	0.005	−2.93	0.023
LTF	−1.73	0.044	−2.56	0.042
VNN2	1.56	0.021	1.55	0.028
TRGV3	1.78	9.18E-04	3.67	0.240*

## Discussion

Histological chorioamnionitis is associated with significant long-term morbidities including asthma and allergic and neurological disorders in preterm and term infants^4–9^. The exact mechanism of increased risk for asthma and allergic disorders in neonates born to mothers with HCA is not well understood. Inflammatory mediators released with HCA may reprogram the developing immune, respiratory, and neurological systems, leading to allergy, asthma, and immunological and neurological disorders^20,21^. To our knowledge, this is the first study reporting the differential gene expression profile in cord blood mononuclear leukocytes in term infants exposed to HCA. Our data indicate that the key genes regulating inflammatory, immune, respiratory, and neurological pathways are differentially expressed in cord blood leukocytes from term infants born to mothers with HCA.

Based on fold change, the top up-regulated genes included chemokine receptor CCR2, T-cell receptor gamma variable-3 (TRGV3), and TRGV4. CCR2 is an important chemokine receptor that induces macrophage and monocytes recruitment to sites of inflammation^22^. CCR2 is up-regulated on T-helper cells in an animal model of allergic asthma and blocking of CCR2 suppressed airway inflammation^18^.The top down-regulated genes included HBEGF, LTF, CAMP, and NR4A3. HBEGF is a potent vasodilator of intestinal microvasculature, and supplementation of HBEGF reduced intestinal injury in animal models of necrotizing enterocolitis (NEC)^23–25^.

*In utero* exposure to HCA leads to fetal inflammatory response syndrome. Understanding the effects of the fetal inflammatory response on the programming of immune, respiratory, and neurological systems in neonates could lead to new ways of modulating these responses and improving long-term consequences. We have identified several key genes involved in immune function, inflammation, and lung development that are expressed differentially after exposure to HCA. These candidate genes are likely to play a critical role in long-term consequences including bronchopulmonary dysplasia (BPD), asthma, allergy, and immunological disorders in neonates exposed to HCA. This warrants further functional investigation.

We report that several important genes involved in the immune system are up-regulated in cord blood mononuclear cells after exposure to HCA. RTKN2 has anti-apoptotic properties, is highly expressed in T-cells, and plays a role as a key signaling switch for regulation of genes involved in T-cell survival^26^. RTKN2 may prolong survival of inflammatory cells and contribute to an exaggerated inflammatory response in neonates exposed to HCA. CD99 is a cell surface protein involved in cell adhesion, migration, differentiation, and apoptosis, and influences processes associated with inflammation and immune response^27^. GIMAP family members play a role in T-helper cell differentiation^28^. GIMAP4 and GIMAP5 have a role in T-cell survival. GIMAP7 is also a regulator of lymphocyte survival and homeostasis^29^. NR4A3, a gene involved in protein coding that regulates Treg cell development through activation of FoxP3, and which may have potential for therapeutic target for immune disorders, was down-regulated^30^. NR4A3 also positively regulates neutrophil survival and homeostasis^31^.

Our data indicate that several key pro-inflammatory mediators are down-regulated in cord blood mononuclear leukocytes after exposure to HCA. The down-regulation of a few pro-inflammatory mediators likely is a modulating mechanism for the exaggerated inflammatory response induced by HCA. The inflammatory mediators downregulated by HCA include PDE4B, CXCL1, TARM1, and IRAK2. PDE4B degrades cyclic AMP, a second messenger involved in inflammatory process. Selective inhibition of PDE4B is a therapeutic target for inflammatory and allergic disorders^32^. CXCL1 is a potent neutrophil chemoattractant that plays a role in the development of BPD^33^. IRAK2 forms a Myddosome complex with Myd88 and IRAK4, activating downstream NF-kB and MAPKs P38 and JNK, leading to inflammatory response^34^. The roles of these pro-inflammatory mediators downregulated by exposure to HCA need further investigation.

We also report that several key genes involved in lung development, BPD, and asthma are expressed differentially in cord blood mononuclear cells after exposure to HCA. Exposure to HCA upregulates CHRNA7, a key mediator of the biological effects of nicotine. CHRNA7 plays a critical role in lung development as well in the pathogenesis of asthma^35,36^, and inhibition of CHRNA7 is an important therapeutic target for asthma^36^. Amphiregulin (AREG) is a member of the epidermal growth factor family and contributes to the regulation of cell proliferation. Recombinant AREG suppresses epithelial cell apoptosis in LPS-induced lung injury in mice^37^. Down-regulation of AREG by HCA may contribute to the development of BPD by increasing epithelial cell apoptosis. CAMP plays a critical role in innate immunity against invasive bacterial infections. CAMP is an important host defense against respiratory pathogens. Suppression of CAMP by HCA may increase the risk for recurrent respiratory infections in neonates exposed to HCA. Ramos-Martinez *et al*. showed that treatment with vitamin D reduced respiratory infections in patients with asthma, and this effect was mediated by increase in CAMP^38^. LTF is an important mucosal antimicrobial protein that is also downregulated in neonates exposed to HCA. Revenis showed that the level of LTF was lower in tracheal aspirate samples from premature infants who developed BPD^39^. Additionally, LTF can down-regulate allergic airway inflammation in asthma^40^. In an animal model of allergic rhinitis, LTF administration in the nostril alleviated allergic rhinitis and its mechanisms^41^. NFIL3 is the most strongly induced transcription factor by IL-4 stimulation and is a critical regulator of IgE production and airway hyper-responsiveness^42,43^. The target genes (SNAI1, ZNF331, Zc3h12a) for the canonical Wnt/B-catenin pathway are down-regulated after exposure to HCA. These target genes of Wnt/B-catenin pathways play a role in the pathogenesis of asthma^44–46^. Peng *et al*. demonstrated that Zc3h12a-knockout mice have severe spontaneous lung inflammation with an increase in IL-5- and IL-13-producing cells in the lung^45^. ZNF331 was expressed differentially in bronchial alveolar lavage cells from patients with asthma^46^. PTEN regulates airway smooth muscle contraction in allergic asthma^47^. Epithelial-mesenchymal transition (EMT) accounts for accumulation of subepithelial mesenchymal cells and contributes to airway hyper-responsiveness and remodeling^48^. Up-regulating expression of PTEN inhibits EMT and may be protective on airway modeling in asthma and BPD. PTGS2/COX2 increases lung inflammation and impairs lung development^33,49^. HBEGF overexpression is associated with airway remodeling and asthma^50^. Interestingly, we found that expression of PTGS2 and HBEGF was down-regulated in cord blood mononuclear cells after exposure to HCA. HBEGF was also downregulated in lung tissue after intra-amniotic LPS administration in an animal model of HCA^51^. The role of PTGS2 and HBEGF on lung development and lung inflammation needs further investigation.

Infants and children born to mothers with HCA are at not only an increased risk for development of inflammatory, immune, and allergic diseases, but they are also predisposed to abnormal neurodevelopmental disorders^7–9^. Our pathway analysis indicates that exposure to HCA affects genes involved in nervous system development, neurological disease, behavioral, developmental, and psychological disorders. Allard *et al*. showed that female rats exposed to *in utero* placental inflammation showed hyperactive behavior after puberty^52^. IPA analysis of our data indicates that HCA differentially expressed genes in the IL-17 signaling pathway, IL-17A signaling in airway cells, and IL-17A signaling in fibroblasts. Recent findings suggests that IL-17A in the fetal and maternal inflammatory response leads to fetal brain injury and neurological sequelae including cerebral palsy and potentially autism, schizophrenia, and multiple sclerosis later in life^53^.

Our study has several strengths. This is the first study reporting differential gene expression in cord blood from term neonates exposed to HCA. A similar study reported differential gene expression after exposure to HCA in preterm infants using whole blood, which does not allow distinction of the relative contribution of leukocytes. Our study is unique as global gene expression was performed in cord blood mononuclear leukocytes, not in whole blood. Further, our global gene expression data were validated by Real-Time PCR for the identified target genes. Six out of seven genes validated with Real-Time PCR showed significant change in the same direction as microarray data, with the seventh gene displaying trends in the same direction. Neonates are vulnerable subjects, and drawing a large amount of blood from them is ethically unacceptable. By using umbilical cord blood, we were able to collect a large amount of blood non-invasively without additional risks to the neonates.

Our study also has several limitations. Our sample size of 10 neonates is small, but similar sample sizes have been used commonly in studies investigating differential gene expression using microarray^12,54^. There is an inherent chance of finding differences in the gene expression due to multiple comparison; however, our results were validated by using Real-Time PCR.

In conclusion, HCA induces differential gene expression in cord blood mononuclear leukocytes from term neonates. We found novel genes and pathways associated with exposure to HCA, including key genes regulating inflammatory, immune, respiratory and neurological pathways. This differential gene expression may contribute to inflammatory, immunological, and neurological disorders in neonates exposed to HCA. Future studies can further validate differential expression of target genes in a larger cohort of neonates exposed to HCA. Our data may lead to understanding the role of key genes and pathways identified in this study on the long-term sequelae related to exposure to HCA. Functional studies on the identified genes and pathways could lead to the development of potential markers for the diseases caused by exposure to HCA and possible therapy to prevent those complications.

## Methods

### Ethical approval: human study protocol and institutional biosafety approvals

All human protocols and procedures described in this study were approved by the Institutional Review Board of Thomas Jefferson University Hospital. All experiments performed in this study were approved and in accordance with the Nemours Institutional Biosafety Committee. The Institutional Review Board waived informed consent as this study was performed on discarded blood and placental tissue samples.

### Study Design

This is a prospective observational study that examines differential gene expression in mononuclear leukocytes isolated from cord blood of term infants born to mothers with HCA. Cord blood and fetal membranes were collected from term neonates (37–40 weeks of gestation). Exclusion criteria were maternal infections other than HCA and complications of pregnancy including diabetes, hypertension, major congenital/chromosomal anomalies, and intrauterine growth restriction.

### Cord blood collection and isolation of leukocytes

At the time of delivery, the umbilical cord was disinfected and cut at the placental side of the clamp. Cord blood was collected in sterile EDTA tubes, mixed thoroughly, and checked for blood clots. Mononuclear leukocytes were isolated by Ficoll-Paque Plus density gradient (GE Healthcare Biosciences, Pittsburgh, PA), following the manufacturer’s protocol and the method of Normann *et al*.^55^. In brief, collected blood was diluted with an equal amount of phosphate buffered saline (PBS). 1 ml of PBS was mixed thoroughly with 16 ml of Ficoll-Paque plus and used as density gradient. 25 to 30 ml of diluted blood was layered on top of the Ficoll mixture and centrifuged at 400xg for 30 minutes. The upper plasma layer was collected and centrifuged to remove platelets. The cellular interface was then collected in a separate tube and washed with PBS containing 1 mM EDTA. Cells were washed with autologous plasma to remove platelets. Finally, the mononuclear leukocytes were washed with PBS, and 2 million packed cells per vial were saved at −80 °C for RNA isolation and microarray analysis.

### Fetal membrane collection, processing, staining, and diagnosis of HCA

Small pieces of fetal membranes tissue were washed with cold PBS and fixed in 10% neutral buffered formalin for 24–48 hours. Membrane pieces were processed and paraffin embedded in Histoplast LP (Thermo Fisher Scientific, Fremont, CA). Samples were sectioned at 5 µm on a Leica RM2255 microtome (Leica, Buffalo Grove, IL) and floated onto Superfrost® Plus slides (Thermo Fisher Scientific, Fremont, CA). The sections were heat immobilized for 60 minutes at 60 °C and were subsequently equilibrated to room temperature prior to staining. The slides were placed on Tissue-Tek Prizma stainer (Sakura Finetek USA, Torrance, CA) and were deparaffinized with xylene, hydrated through a graded series of alcohols, and hydrated to water. Finally, the slides were stained with Harris hematoxylin (BBC Biomedical, Mount Vernon, WA) and eosin (Acros Organics, Cole-Parmer, Vernon Hills, IL) and were dehydrated, cleared, and mounted in Permount® (Thermo Fisher Scientific, Fremont, CA). Tissue samples were processed by the Nemours Histochemistry and Tissue Processing Core using standard workflows and operating procedures. The stained slides were examined by a pathologist (JC) and classified either as HCA (placental membranes score ≥ stage 1) or no HCA (no histological inflammatory changes in fetal membranes)^56^.

### RNA Isolation and Gene Expression Study

Total RNA was isolated using Qiagen miRNeasy Mini Kit (Qiagen, Germantown, MD). RNA was quantified on a Nanodrop ND-2000 spectrophotometer (Thermo Fisher Scientific, Waltham, MA), and quality was assessed by an Agilent 2200 TapeStation (Agilent Technologies, Palo Alto, CA). 100 ng of RNA was used from each sample to prepare fragmented biotin-labeled cDNA by GeneChip WT PLUS reagent kit (Affymetrix, Santa Clara, CA). The Affymetrix gene chips and Human Transcriptome Array 2.0 were hybridized with 5 µg of fragmented biotin-labeled cDNA in 220 µl hybridization cocktail, followed by target denaturation at 99 °C for 5 min and then 45 °C for 5 min. Hybridization was performed for 16 hours at 45 °C with a rotation of 60 rpm. An Affymetrix GeneChip hybridization wash and stain kit was used to wash and stain the arrays in GeneChip Fluidic Station 450. Chips were scanned on an Affymetrix GeneChip Scanner 3000 using Command Console Software (Thermo Fisher Scientific, Waltham, MA). Expression Console Software v1.4.1 was used to perform quality control.

### Real-Time PCR

Selected genes from microarray data were validated by Quantitative Real Time PCR with TaqMan Gene Expression Assay Mix from Applied Biosystems (Applied Biosystems, Foster City, CA) following the manufacturer’s protocol. In brief, 200 ng of total RNA was reverse transcribed with a High-Capacity cDNA RT kit (Applied Biosystems, Foster City, CA) in a total reaction volume of 20 µl. Real-Time PCR was performed in duplicate wells with 0.5 µl of cDNA preparation using TaqMan Universal PCR Master Mix and TaqMan Gene Expression Assay mixes specific for different genes. Actin B was used as a housekeeping gene, and data were collected by Quant Studio 12K Flex Thermal Cycler (Applied Biosystems, Foster City, CA).

### Statistical Analysis

Expression Console Software was used to generate a Chp file from Affymetrix after sst-rma normalization. The HCA Group was compared with the control group using Transcriptome array console software v 1.4.1 (Thermo Fisher Scientific, Waltham, MA). Genes with fold change ≥1.5 and p < 0.05 were identified as differentially expressed. Student t-test was performed for comparison of the two groups. Gene expression data are available at the Gene Expression Omnibus (GEO) database of the NIH under accession number GSE120855. Data were analyzed through the use of IPA (QIAGEN Inc., https://www.qiagenbioinformatics.com/products/ingenuitypathway-analysis)^19^. Real-Time data were compared using Student t-test or Wilcoxon signed rank test between the HCA and control group.

## Supplementary information

Supplementary Tables
