# Supplementary material for: Histological Chorioamnionitis Induces Differential Gene Expression in Human Cord Blood Mononuclear Leukocytes from Term Neonates
Source: Sci Rep. 2019 Apr 10;9:5862. doi: 10.1038/s41598-019-42205-x (PMC6458165; doi:10.1038/s41598-019-42205-x)
Supplement: Supplementary file 1 — Supplementary Tables [file 41598_2019_42205_MOESM1_ESM.pdf]

# Histological Chorioamnionitis Induces Differential Gene Expression in Human Cord Blood Mononuclear Leukocytes from Term Neonates

Suhita Gayen nee' Betal[1], Swati Murthy[1], Michael Favara[1], Gina Fong[1], Joanna SY Chan[2], Sankar Addya[3], Thomas H Shaffer[1], Jay Greenspan[1], Vineet Bhandari[4], Irfan Rahman[5], Zubair H. Aghai[1]*

[1]Neonatology, Thomas Jefferson University/Nemours, Philadelphia, PA, USA

[2]Department of Pathology, Thomas Jefferson University, Philadelphia, PA

[3]Laboratory of Cancer Genomics, Thomas Jefferson University, Philadelphia, PA

[4]Section of Neonatology, Department of Pediatrics, St. Christopher's Hospital for Children, Drexel University College of Medicine, Philadelphia, PA, USA.

[5]Department of Environmental Medicine, University of Rochester Medical Center, Rochester, NY, United States.

*Corresponding author:

Zubair H Aghai, MD

Professor of Pediatrics

Thomas Jefferson University

Nemours at TJU

Philadelphia, PA, USA

zaghai@nemours.org

**Table 1: Differentially upregulated probeID or genes after exposure to histological chorioamanionitis:**

| ProbeID | Gene Symbol | CHORIO Group Average Expression | Control Group Average Expression | Fold Change | P-value |
|---|---|---|---|---|---|
| TC17001604.hg.1 | **LRRC37A4P** | 1389.16 | 344.89 | **4.01** | 0.0245 |
| TC04002938.hg.1 | **LOC285505** | 364.56 | 136.24 | **2.68** | 0.012 |
| TC07000288.hg.1 | **LINC01061** | 1652.00 | 643.59 | **2.58** | 0.0117 |
| TC03000255.hg.1 | **CCR2** | 765.36 | 304.44 | **2.51** | 0.0417 |
| TC04001682.hg.1 | **FAM198B** | 643.59 | 256.00 | **2.5** | 0.0167 |
| TC17000604.hg.1 | **LRRC37A2** | 13216.02 | 5556.65 | **2.37** | 0.0312 |
| TC07001299.hg.1 | **TRGV4** | 140.07 | 60.13 | **2.32** | 0.0002 |
| TC07001300.hg.1 | **TRGV3** | 179.77 | 79.89 | **2.24** | 4.50E-05 |
| TC04002939.hg.1 | **TMEM144** | 240.52 | 112.99 | **2.12** | 0.0173 |
| TC17001603.hg.1 | **LRRC37A4P; LRRC37A2** | 20594.91 | 9946.68 | **2.08** | 0.0478 |
| TC04001501.hg.1 | **LINC01061** | 25709.25 | 13216.02 | **1.94** | 0.0257 |
| TC05001269.hg.1 | **MIR580** | 77.17 | 40.50 | **1.91** | 0.0005 |
| TC15001206.hg.1 | **MIR3942** | 278.20 | 151.17 | **1.84** | 0.0293 |
| TC10000103.hg.1 | **LOC101928524** | 304.44 | 167.73 | **1.81** | 0.0052 |
| TC12003271.hg.1 | **KLRC3** | 1458.23 | 809.00 | **1.8** | 0.0433 |
| TC19000976.hg.1 | **LINC01002** | 53602.03 | 30152.71 | **1.77** | 0.036 |
| TC19000978.hg.1 | **LOC730378** | 211456.30 | 121449.75 | **1.75** | 0.0089 |
| TC17_ctg5_hap1000003.hg.1 | **ARL17A** | 1758.34 | 1009.90 | **1.74** | 0.0499 |
| TC05000981.hg.1 | **HRH2** | 1734.13 | 1002.93 | **1.74** | 0.0233 |
| TC04000209.hg.1 | **DTHD1** | 215.27 | 124.50 | **1.73** | 0.0326 |
| TC01002022.hg.1 | **OR2L1P** | 123.64 | 71.51 | **1.73** | 0.0179 |
| TC01000005.hg.1 | **LINC01001** | 34397.12 | 20452.65 | **1.67** | 0.0283 |
| TC13000389.hg.1 | **LINC00676** | 54.95 | 33.13 | **1.66** | 0.0229 |
| TC01002066.hg.1 | **LINC01001** | 80684.28 | 48644.87 | **1.66** | 0.0297 |
| TC07003403.hg.1 | **LOC401320** | 3082.75 | 1858.60 | **1.65** | 0.0099 |
| TC05003443.hg.1 | **LINC01001** | 51418.50 | 31433.17 | **1.64** | 0.0416 |
| TC11003501.hg.1 | **LINC01001** | 31433.17 | 19215.73 | **1.63** | 0.0363 |
| TC22000031.hg.1 | **LOC727983** | 942.27 | 580.04 | **1.62** | 0.0224 |
| TC15001137.hg.1 | **CHRNA7** | 33.36 | 20.68 | **1.61** | 0.0201 |
| TC18000436.hg.1 | **DSC2** | 1520.15 | 948.83 | **1.61** | 0.0221 |
| TC07001003.hg.1 | **GIMAP7** | 4482.23 | 2778.33 | **1.61** | 0.0086 |

| ProbeID | Gene Symbol | CHORIO Group Average Expression | Control Group Average Expression | Fold Change | P-value |
|---|---|---|---|---|---|
| TC10001324.hg.1 | **RTKN2** | 153.28 | 96.34 | **1.59** | 0.0294 |
| TC09000265.hg.1 | **RP11-187C18.2** | 729.11 | 464.65 | **1.57** | 0.0034 |
| TC0Y000014.hg.1 | **CD99** | 2646.74 | 1698.45 | **1.56** | 0.0121 |
| TC02001750.hg.1 | **CYP1B1** | 448.82 | 288.01 | **1.56** | 0.0394 |
| TC0X000014.hg.1 | **CD99** | 1520.15 | 975.50 | **1.55** | 0.0102 |
| TC10000625.hg.1 | **PTEN** | 694.58 | 448.82 | **1.55** | 0.0275 |
| TC07003404.hg.1 | **LOC401320** | 1296.13 | 843.36 | **1.54** | 0.0266 |
| TC17001612.hg.1 | **ARL17B; ARL17A** | 2665.15 | 1746.20 | **1.53** | 0.0215 |
| TC07003399.hg.1 | **GIMAP1-GIMAP5; GIMAP5; GIMAP1** | 989.12 | 648.07 | **1.53** | 0.0155 |
| TC07001004.hg.1 | **GIMAP4** | 5595.30 | 3691.52 | **1.52** | 0.0482 |
| TC12000873.hg.1 | **ADAM1A** | 487.75 | 324.03 | **1.51** | 0.0085 |
| TC01004036.hg.1 | **LINC01347** | 28924.41 | 19215.73 | **1.51** | 0.0473 |
| TC01000016.hg.1 | **LOC100287934** | 1209.34 | 803.41 | **1.51** | 0.001 |
| TC11000314.hg.1 | **LINC00294** | 298.17 | 198.09 | **1.5** | 0.0458 |
| TC02004349.hg.1 | **LOC105374809** | 229.13 | 74.03 | **3.08** | 0.0077 |
| TC04002292.hg.1 | **LOC285505** | 385.34 | 135.30 | **2.84** | 0.0083 |
| TC07002675.hg.1 | **GIMAP4** | 52498.92 | 23010.42 | **2.29** | 0.0118 |
| TC19002275.hg.1 | **LINC01002** | 42642.37 | 21469.49 | **1.99** | 0.0435 |
| TC15002743.hg.1 | **PRKXP1** | 30786.28 | 15608.02 | **1.98** | 0.0245 |
| TC04002690.hg.1 | **LINC01061** | 12161.22 | 6888.62 | **1.77** | 0.0129 |
| TC06003855.hg.1 | **VNN2** | 14164.58 | 8306.36 | **1.7** | 0.007 |
| TC01005171.hg.1 | **OR2L1P** | 106.89 | 63.56 | **1.68** | 0.0211 |
| TC11002977.hg.1 | **LINC01001** | 50360.33 | 30786.28 | **1.63** | 0.0419 |
| TC04002162.hg.1 | **LOC729218** | 18561.17 | 11505.21 | **1.62** | 0.0264 |
| TC07002674.hg.1 | **GIMAP7** | 4182.07 | 2610.30 | **1.6** | 0.0263 |
| TC07002869.hg.1 | **LOC401320** | 1128.35 | 704.28 | **1.6** | 0.0116 |
| TC10001807.hg.1 | **WDR37** | 1398.83 | 903.89 | **1.54** | 0.0184 |
| TC12002570.hg.1 | **ADAM1A** | 451.94 | 296.11 | **1.53** | 0.0034 |
| TC03002770.hg.1 | **LRCH3** | 809.00 | 530.06 | **1.53** | 0.049 |
| TC01003858.hg.1 | | 1458.23 | 205.07 | **7.1** | 0.0331 |
| TC01003860.hg.1 | | 21920.61 | 7332.05 | **2.99** | 0.0013 |
| TC01003859.hg.1 | | 5148.73 | 1758.34 | **2.93** | 0.0058 |
| TC07000290.hg.1 | | 5148.73 | 1758.34 | **2.93** | 0.0058 |

| ProbeID | Gene Symbol | CHORIO Group Average Expression | Control Group Average Expression | Fold Change | P-value |
|---|---|---|---|---|---|
| TC07000368.hg.1 | | 5148.73 | 1758.34 | **2.93** | 0.0058 |
| TC04000607.hg.1 | | 10085.54 | 3541.14 | **2.85** | 0.0301 |
| TC07001400.hg.1 | | 8599.28 | 3104.19 | **2.77** | 0.0044 |
| TC01003861.hg.1 | | 7231.10 | 2628.46 | **2.76** | 0.0023 |
| TC01001880.hg.1 | | 8902.53 | 3258.52 | **2.73** | 0.0056 |
| TC07001379.hg.1 | | 8902.53 | 3258.52 | **2.73** | 0.0056 |
| TC07001411.hg.1 | | 8902.53 | 3258.52 | **2.73** | 0.0056 |
| TC07001421.hg.1 | | 8902.53 | 3258.52 | **2.73** | 0.0056 |
| TC05000308.hg.1 | | 5220.60 | 1924.14 | **2.72** | 0.0444 |
| TC05000320.hg.1 | | 5220.60 | 1924.14 | **2.72** | 0.0444 |
| TC05000486.hg.1 | | 5220.60 | 1924.14 | **2.72** | 0.0444 |
| TC05001199.hg.1 | | 5220.60 | 1924.14 | **2.72** | 0.0444 |
| TC05001633.hg.1 | | 5220.60 | 1924.14 | **2.72** | 0.0444 |
| TC20000159.hg.1 | | 10369.08 | 3848.29 | **2.69** | 0.0443 |
| TC01001878.hg.1 | | 1652.00 | 643.59 | **2.58** | 0.0117 |
| TC07000366.hg.1 | | 1652.00 | 643.59 | **2.58** | 0.0117 |
| TC07001381.hg.1 | | 1652.00 | 643.59 | **2.58** | 0.0117 |
| TC07001402.hg.1 | | 1652.00 | 643.59 | **2.58** | 0.0117 |
| TC07001412.hg.1 | | 1652.00 | 643.59 | **2.58** | 0.0117 |
| TC07001423.hg.1 | | 1652.00 | 643.59 | **2.58** | 0.0117 |
| TC01001879.hg.1 | | 18305.63 | 7181.15 | **2.56** | 0.0019 |
| TC07000289.hg.1 | | 18305.63 | 7181.15 | **2.56** | 0.0019 |
| TC07000367.hg.1 | | 18305.63 | 7181.15 | **2.56** | 0.0019 |
| TC07001380.hg.1 | | 18305.63 | 7181.15 | **2.56** | 0.0019 |
| TC07001422.hg.1 | | 18305.63 | 7181.15 | **2.56** | 0.0019 |
| TC04001593.hg.1 | | 380.04 | 149.09 | **2.54** | 0.0317 |
| TC07000292.hg.1 | | 3565.78 | 1428.22 | **2.51** | 0.0213 |
| TC07000291.hg.1 | | 2120.22 | 873.10 | **2.43** | 0.0059 |
| TC07001378.hg.1 | | 2120.22 | 873.10 | **2.43** | 0.0059 |
| TC07001399.hg.1 | | 2120.22 | 873.10 | **2.43** | 0.0059 |
| TC07001410.hg.1 | | 2120.22 | 873.10 | **2.43** | 0.0059 |
| TC10000923.hg.1 | | 533.74 | 225.97 | **2.36** | 0.0218 |
| TC07001401.hg.1 | | 24833.50 | 10734.74 | **2.31** | 0.0023 |

| ProbeID | Gene Symbol | CHORIO Group Average Expression | Control Group Average Expression | Fold Change | P-value |
|---|---|---|---|---|---|
| TC15001366.hg.1 | | 52.35 | 24.25 | **2.16** | 0.005 |
| TC16001268.hg.1 | | 8659.09 | 4039.61 | **2.14** | 0.0273 |
| TC01001881.hg.1 | | 14362.31 | 6984.79 | **2.06** | 0.0427 |
| TC01003854.hg.1 | | 14362.31 | 6984.79 | **2.06** | 0.0427 |
| TC0Y000089.hg.1 | | 15935.98 | 7912.95 | **2.02** | 0.0427 |
| TC0Y000215.hg.1 | | 15935.98 | 7912.95 | **2.02** | 0.0427 |
| TC0X001157.hg.1 | | 2646.74 | 1314.23 | **2.02** | 0.0035 |
| TC10000182.hg.1 | | 1541.37 | 765.36 | **2.02** | 0.0027 |
| TC04001681.hg.1 | | 61.82 | 31.34 | **1.96** | 0.0033 |
| TC08000385.hg.1 | | 26432.04 | 13777.25 | **1.91** | 0.0223 |
| TC01003997.hg.1 | | 2225.63 | 1168.14 | **1.91** | 0.0358 |
| TC01001143.hg.1 | | 74.54 | 39.12 | **1.9** | 0.0385 |
| TC01003428.hg.1 | | 74.54 | 39.12 | **1.9** | 0.0385 |
| TC13000535.hg.1 | | 74.54 | 39.12 | **1.9** | 0.0385 |
| TC17001431.hg.1 | | 74.54 | 39.12 | **1.9** | 0.0385 |
| TC10000501.hg.1 | | 20.68 | 11.00 | **1.88** | 9.89E-05 |
| TC04000892.hg.1 | | 935.76 | 494.56 | **1.88** | 0.0329 |
| TC04001002.hg.1 | | 155.42 | 83.87 | **1.86** | 0.0178 |
| TC21000099.hg.1 | | 229.13 | 124.50 | **1.85** | 0.046 |
| TC17001365.hg.1 | | 2998.45 | 1675.06 | **1.8** | 0.0014 |
| TC10001099.hg.1 | | 71.51 | 40.22 | **1.78** | 0.0305 |
| TC09000313.hg.1 | | 54.57 | 30.70 | **1.78** | 0.0178 |
| TC12000053.hg.1 | | 21.41 | 12.13 | **1.77** | 0.0098 |
| TC17001605.hg.1 | | 1910.85 | 1089.92 | **1.76** | 0.0372 |
| TC19000979.hg.1 | | 105728.15 | 61572.56 | **1.72** | 0.0209 |
| TC06001135.hg.1 | | 666.29 | 393.44 | **1.7** | 0.0341 |
| TC05000244.hg.1 | | 2916.45 | 1722.16 | **1.69** | 0.0348 |
| TC12001370.hg.1 | | 76.11 | 45.25 | **1.68** | 0.0105 |
| TC11001725.hg.1 | | 104.69 | 62.25 | **1.68** | 0.0066 |
| TC01003098.hg.1 | | 100.43 | 60.13 | **1.67** | 0.0065 |
| TC01003103.hg.1 | | 100.43 | 60.13 | **1.67** | 0.0065 |
| TC19000267.hg.1 | | 75.58 | 44.94 | **1.67** | 0.0008 |
| TC10000083.hg.1 | | 2005.85 | 1217.75 | **1.65** | 0.011 |
| TC01001877.hg.1 | | 128374.58 | 78477.96 | **1.63** | 0.02 |

| ProbeID | Gene Symbol | CHORIO Group Average Expression | Control Group Average Expression | Fold Change | P-value |
|---|---|---|---|---|---|
| TC01003862.hg.1 | | 128374.58 | 78477.96 | **1.63** | 0.02 |
| TC06001206.hg.1 | | 128374.58 | 78477.96 | **1.63** | 0.02 |
| TC07000287.hg.1 | | 128374.58 | 78477.96 | **1.63** | 0.02 |
| TC07001403.hg.1 | | 128374.58 | 78477.96 | **1.63** | 0.02 |
| TC07001413.hg.1 | | 128374.58 | 78477.96 | **1.63** | 0.02 |
| TCUn_gl000227000001.hg.1 | | 128374.58 | 78477.96 | **1.63** | 0.02 |
| TC06000434.hg.1 | | 39.67 | 24.76 | **1.6** | 0.0004 |
| TC11000334.hg.1 | | 8422.31 | 5330.30 | **1.59** | 0.0167 |
| TC17000111.hg.1 | | 94.35 | 60.13 | **1.58** | 0.0133 |
| TC07000365.hg.1 | | 35857.82 | 22851.48 | **1.58** | 0.0215 |
| TC07001382.hg.1 | | 35857.82 | 22851.48 | **1.58** | 0.0215 |
| TC07001424.hg.1 | | 35857.82 | 22851.48 | **1.58** | 0.0215 |
| TC05001349.hg.1 | | 240.52 | 154.34 | **1.56** | 0.0353 |
| TC15001432.hg.1 | | 103.97 | 67.18 | **1.55** | 0.0314 |
| TC01001404.hg.1 | | 51.63 | 33.59 | **1.54** | 0.0227 |
| TC01002198.hg.1 | | 93.70 | 60.97 | **1.54** | 0.0039 |
| TC01001840.hg.1 | | 2368.90 | 1562.89 | **1.52** | 0.0148 |
| TC10000525.hg.1 | | 25.81 | 16.91 | **1.52** | 0.0254 |
| TC05002421.hg.1 | | 1924.14 | 820.30 | **2.35** | 0.0024 |
| TC07002898.hg.1 | | 349.71 | 152.22 | **2.3** | 1.66E-06 |
| TC05002667.hg.1 | | 522.76 | 250.73 | **2.08** | 0.0082 |
| TC21000653.hg.1 | | 257.78 | 128.00 | **2.01** | 0.0354 |
| TC07002667.hg.1 | | 99.04 | 49.18 | **2.01** | 0.0401 |
| TC07002607.hg.1 | | 171.25 | 86.82 | **1.97** | 0.0026 |
| TC17002302.hg.1 | | 209.38 | 112.21 | **1.88** | 0.0277 |
| TC17002671.hg.1 | | 1845.76 | 982.29 | **1.87** | 0.044 |
| TC10002194.hg.1 | | 867.07 | 471.14 | **1.84** | 0.0058 |
| TC01004101.hg.1 | | 113316.62 | 63303.60 | **1.79** | 0.0157 |
| TC04002413.hg.1 | | 182.28 | 102.54 | **1.78** | 0.045 |
| TC06002765.hg.1 | | 147.03 | 83.87 | **1.76** | 0.0012 |
| TC0X002129.hg.1 | | 3795.30 | 2164.77 | **1.74** | 0.0008 |
| TC17002157.hg.1 | | 151.17 | 88.03 | **1.72** | 0.0306 |
| TC04002164.hg.1 | | 44146.15 | 25709.25 | **1.71** | 0.0202 |
| TC01005080.hg.1 | | 1082.39 | 643.59 | **1.69** | 0.0147 |

| ProbeID | Gene Symbol | CHORIO Group Average Expression | Control Group Average Expression | Fold Change | P-value |
|---|---|---|---|---|---|
| TC04002324.hg.1 | | 44.32 | 26.35 | **1.69** | 0.0432 |
| TC13001493.hg.1 | | 38.85 | 22.94 | **1.69** | 0.0002 |
| TC04002163.hg.1 | | 5832.91 | 3468.27 | **1.68** | 0.0206 |
| TC04002143.hg.1 | | 326.29 | 195.36 | **1.68** | 0.0067 |
| TC01006114.hg.1 | | 121.94 | 74.03 | **1.65** | 0.0164 |
| TC07002897.hg.1 | | 106.15 | 65.34 | **1.63** | 0.0002 |
| TC02004043.hg.1 | | 210.84 | 129.79 | **1.63** | 0.0282 |
| TC10002017.hg.1 | | 16270.83 | 10015.87 | **1.63** | 0.0223 |
| TC01006151.hg.1 | | 34159.52 | 21321.18 | **1.61** | 0.0389 |
| TC20001566.hg.1 | | 873.10 | 541.19 | **1.61** | 0.0124 |
| TC01004694.hg.1 | | 1105.13 | 699.41 | **1.59** | 0.0396 |
| TC01004960.hg.1 | | 179.77 | 114.56 | **1.58** | 0.0148 |
| TC01004838.hg.1 | | 317.37 | 200.85 | **1.58** | 0.0192 |
| TC01006140.hg.1 | | 25709.25 | 16384.00 | **1.57** | 0.0187 |
| TC14001804.hg.1 | | 290.02 | 184.82 | **1.57** | 0.0178 |
| TC18000918.hg.1 | | 233.94 | 149.09 | **1.57** | 0.0039 |
| TC09001978.hg.1 | | 210.84 | 136.24 | **1.55** | 0.0158 |
| TC17002484.hg.1 | | 167.73 | 108.38 | **1.55** | 0.0293 |
| TC17002059.hg.1 | | 165.42 | 106.15 | **1.55** | 0.0079 |
| TC10001946.hg.1 | | 1031.12 | 670.92 | **1.54** | 0.0039 |
| TC06003586.hg.1 | | 26.35 | 17.15 | **1.54** | 0.0272 |
| TC07003176.hg.1 | | 266.87 | 173.65 | **1.54** | 0.0087 |
| TC09002409.hg.1 | | 461.44 | 298.17 | **1.54** | 0.0101 |
| TC15002562.hg.1 | | 132.51 | 86.82 | **1.53** | 0.0277 |
| TC16001990.hg.1 | | 1251.98 | 820.30 | **1.53** | 0.0254 |
| TC13001186.hg.1 | | 218.27 | 143.01 | **1.53** | 0.0465 |
| TC01005193.hg.1 | | 68794.23 | 44762.41 | **1.53** | 0.0318 |
| TC01005474.hg.1 | | 270.60 | 178.53 | **1.52** | 0.0238 |
| TC07002294.hg.1 | | 1438.15 | 942.27 | **1.52** | 0.0181 |
| TC10001901.hg.1 | | 2241.11 | 1468.37 | **1.52** | 0.0123 |
| TC17002234.hg.1 | | 588.13 | 390.72 | **1.51** | 0.0073 |
| TC12002436.hg.1 | | 41.07 | 27.10 | **1.51** | 0.0002 |
| TC07002647.hg.1 | | 182.28 | 121.10 | **1.51** | 0.0276 |

**Table 2: Differentially downregulated probeID or genes after exposure to histological chorioamnionitis:**

| ProbeID | Gene Symbol | CHORIO Group Average Expression | Control Group Average Expression | Fold Change | P-value |
|---|---|---|---|---|---|
| TC01003638.hg.1 | **PTGS2** | 252.48 | 9089.59 | **-35.91** | 0.0205 |
| TC04002953.hg.1 | **AREG** | 313.00 | 1884.54 | **-6.03** | 0.001 |
| TC04002952.hg.1 | **AREG** | 328.56 | 1897.65 | **-5.75** | 0.0009 |
| TC01001749.hg.1 | **G0S2** | 2352.53 | 13034.07 | **-5.54** | 0.008 |
| TC05001854.hg.1 | **HBEGF** | 385.34 | 2005.85 | **-5.18** | 0.0022 |
| TC19001593.hg.1 | **PLAUR** | 1448.15 | 7082.29 | **-4.89** | 0.0138 |
| TC21001069.hg.1 | **SAMSN1** | 3956.48 | 17438.64 | **-4.42** | 0.0257 |
| TC12001216.hg.1 | **OLR1** | 178.53 | 765.36 | **-4.27** | 0.0365 |
| TC03000276.hg.1 | **CAMP** | 657.11 | 2683.69 | **-4.09** | 0.0011 |
| TC09000508.hg.1 | **NR4A3** | 143.01 | 580.04 | **-4.07** | 0.0128 |
| TC03001357.hg.1 | **LTF** | 2683.69 | 9345.14 | **-3.49** | 0.0208 |
| TC03002133.hg.1 | **ATP13A3** | 1618.00 | 5634.22 | **-3.47** | 0.0063 |
| TC10000566.hg.1 | **PPIF** | 1152.06 | 3541.14 | **-3.09** | 0.0226 |
| TC10000629.hg.1 | **LIPN** | 519.15 | 1584.71 | **-3.05** | 0.0234 |
| TC09001325.hg.1 | **NFIL3** | 1192.69 | 3304.00 | **-2.77** | 0.0262 |
| TC05001589.hg.1 | **LUCAT1** | 265.03 | 729.11 | **-2.75** | 0.0057 |
| TC01003629.hg.1 | **IVNS1ABP** | 8422.31 | 21469.49 | **-2.55** | 0.0036 |
| TC01001622.hg.1 | **RGS1** | 1428.22 | 3541.14 | **-2.47** | 0.0042 |
| TC17000117.hg.1 | **KDM6B** | 1200.98 | 2916.45 | **-2.42** | 0.0318 |
| TC19000885.hg.1 | **FCAR** | 2256.70 | 5330.30 | **-2.35** | 0.0023 |
| TC01000733.hg.1 | **PDE4B** | 280.14 | 652.58 | **-2.33** | 0.0125 |
| TC19001241.hg.1 | **ADGRE3** | 680.29 | 1573.76 | **-2.3** | 0.042 |
| TC06000844.hg.1 | **PRDM1** | 263.20 | 600.49 | **-2.28** | 0.006 |
| TC0X000079.hg.1 | **SYAP1** | 968.76 | 2164.77 | **-2.22** | 0.0013 |
| TC02002102.hg.1 | **DUSP2** | 474.41 | 1031.12 | **-2.18** | 0.0153 |
| TC03001302.hg.1 | **CSRNP1** | 617.37 | 1287.18 | **-2.09** | 0.0268 |
| TC0X000112.hg.1 | **SAT1** | 13873.08 | 28924.41 | **-2.08** | 0.0162 |
| TC03000766.hg.1 | **ATP1B3** | 2288.20 | 4705.07 | **-2.06** | 0.0363 |
| TC04000411.hg.1 | **CXCL1** | 112.21 | 229.13 | **-2.05** | 0.0404 |
| TC19001830.hg.1 | **TARM1** | 116.16 | 235.57 | **-2.02** | 0.0059 |
| TC17000983.hg.1 | **MIR22HG; MIR22** | 410.15 | 826.00 | **-2.01** | 0.0006 |
| TC12000414.hg.1 | **NR4A1** | 144.01 | 288.01 | **-2.01** | 0.0174 |
| TC22000648.hg.1 | **OSM** | 68.12 | 134.36 | **-1.97** | 0.0458 |

| ProbeID | Gene Symbol | CHORIO Group Average Expression | Control Group Average Expression | Fold Change | P-value |
|---|---|---|---|---|---|
| TC05001385.hg.1 | **PLK2** | 95.01 | 187.40 | **-1.97** | 0.0396 |
| TC15002005.hg.1 | **CHSY1** | 1686.71 | 3304.00 | **-1.96** | 0.0039 |
| TC10000922.hg.1 | **PTPRE** | 4329.55 | 8364.13 | **-1.93** | 0.0206 |
| TC20000395.hg.1 | **SNAI1** | 47.18 | 91.14 | **-1.93** | 0.0083 |
| TC06002120.hg.1 | **VNN3** | 604.67 | 1168.14 | **-1.93** | 0.039 |
| TC20000844.hg.1 | **MAFB** | 207.94 | 398.93 | **-1.92** | 0.0097 |
| TC05001628.hg.1 | **CHD1** | 8023.41 | 15286.81 | **-1.9** | 0.0413 |
| TC01000950.hg.1 | **AMPD2** | 670.92 | 1269.46 | **-1.89** | 0.0104 |
| TC10000189.hg.1 | **MASTL** | 335.46 | 634.73 | **-1.89** | 0.0091 |
| TC07003301.hg.1 | **PNPLA8** | 1820.35 | 3326.99 | **-1.83** | 0.007 |
| TC19001457.hg.1 | **NFKBID** | 261.38 | 471.14 | **-1.81** | 0.0095 |
| TC01003359.hg.1 | **ETV3** | 1152.06 | 2076.59 | **-1.8** | 0.0099 |
| TC22001424.hg.1 | **APOBEC3A_B; APOBEC3A** | 2149.82 | 3821.70 | **-1.77** | 0.0451 |
| TC19000812.hg.1 | **ZNF331** | 288.01 | 512.00 | **-1.77** | 0.0308 |
| TC01000474.hg.1 | **ZC3H12A** | 256.00 | 451.94 | **-1.76** | 0.0422 |
| TC17000141.hg.1 | **NDEL1** | 3350.13 | 5873.48 | **-1.75** | 0.0038 |
| TC04000678.hg.1 | **NOCT** | 205.07 | 362.04 | **-1.75** | 0.008 |
| TC14000694.hg.1 | **SNORD114-17** | 50.21 | 88.03 | **-1.75** | 0.0121 |
| TC06001498.hg.1 | **TRIM10** | 116.16 | 203.66 | **-1.75** | 0.0443 |
| TC19000274.hg.1 | **CYP4F3** | 202.25 | 352.14 | **-1.74** | 0.0449 |
| TC03000056.hg.1 | **IRAK2** | 164.28 | 284.05 | **-1.74** | 0.048 |
| TC10000413.hg.1 | **DDX21** | 7486.11 | 12854.63 | **-1.71** | 0.036 |
| TC21000506.hg.1 | **SIK1** | 968.76 | 1652.00 | **-1.7** | 0.0182 |
| TC05001570.hg.1 | **CCNH** | 2665.15 | 4451.27 | **-1.68** | 0.0061 |
| TC05001610.hg.1 | **ELL2** | 709.18 | 1192.69 | **-1.68** | 0.0165 |
| TC07001738.hg.1 | **NAMPT** | 67378.47 | 110984.60 | **-1.65** | 0.0367 |
| TC15000111.hg.1 | **SNORD115-42; SNORD115-10** | 91.14 | 151.17 | **-1.65** | 0.0175 |
| TC09000274.hg.1 | **CNTNAP3B** | 40.79 | 66.72 | **-1.63** | 0.026 |
| TC02000183.hg.1 | **FOSL2** | 837.53 | 1370.04 | **-1.63** | 0.0317 |
| TC15000080.hg.1 | **SNORD115-6** | 108.38 | 176.07 | **-1.63** | 0.0198 |
| TC07001304.hg.1 | **FAM183BP** | 116.97 | 187.40 | **-1.6** | 0.0145 |
| TC03000096.hg.1 | **EAF1** | 1128.35 | 1807.78 | **-1.59** | 0.0257 |
| TC19001351.hg.1 | **LOC105372321** | 77.71 | 124.50 | **-1.59** | 0.014 |
| TC06001762.hg.1 | **NFKBIE** | 413.00 | 657.11 | **-1.59** | 0.0351 |

| ProbeID | Gene Symbol | CHORIO Group Average Expression | Control Group Average Expression | Fold Change | P-value |
|---|---|---|---|---|---|
| TC01001720.hg.1 | **CTSE** | 121.94 | 191.34 | **-1.57** | 0.0366 |
| TC05001831.hg.1 | **ETF1** | 2288.20 | 3590.58 | **-1.57** | 0.0124 |
| TC17001118.hg.1 | **MYH10** | 77.71 | 121.94 | **-1.57** | 0.0407 |
| TC07003328.hg.1 | **RABGEF1** | 1530.73 | 2418.67 | **-1.57** | 0.0019 |
| TC04001570.hg.1 | **SLC7A11** | 57.68 | 91.14 | **-1.57** | 0.0048 |
| TC06000532.hg.1 | **CDKN1A** | 385.34 | 596.34 | **-1.55** | 0.0076 |
| TC17000108.hg.1 | **ATP1B2** | 50.56 | 78.25 | **-1.54** | 0.0383 |
| TC0X000238.hg.1 | **TIMP1** | 413.00 | 634.73 | **-1.54** | 0.0184 |
| TC17000119.hg.1 | **CYB5D1** | 88.65 | 135.30 | **-1.53** | 0.0471 |
| TC16001302.hg.1 | **MPHOSPH6** | 418.77 | 634.73 | **-1.51** | 0.0039 |
| TC19001640.hg.1 | **PGLYRP1** | 321.80 | 484.38 | **-1.51** | 0.0324 |
| TC15000093.hg.1 | **SNORD115-21** | 47.84 | 72.00 | **-1.51** | 0.0156 |
| TC01004998.hg.1 | **G0S2** | 4608.24 | 50012.46 | **-10.82** | 0.0054 |
| TC07003113.hg.1 | **DOCK4** | 564.18 | 4240.45 | **-7.51** | 0.0092 |
| TC06003603.hg.1 | **HLA-DQB1** | 315.17 | 2272.40 | **-7.18** | 0.0089 |
| TC02004133.hg.1 | **BRE-AS1** | 149.09 | 975.50 | **-6.55** | 0.036 |
| TC19002226.hg.1 | **ZNF331** | 268.73 | 1060.11 | **-3.94** | 0.023 |
| TC19002237.hg.1 | **FCAR** | 8135.41 | 26801.01 | **-3.3** | 0.0076 |
| TC11003025.hg.1 | **BGLT3** | 78.79 | 240.52 | **-3.06** | 0.0237 |
| TC12002776.hg.1 | **ART4** | 30.06 | 73.52 | **-2.45** | 0.0061 |
| TC06003854.hg.1 | **VNN3** | 935.76 | 2288.20 | **-2.45** | 0.0214 |
| TC17002479.hg.1 | **MYH10** | 71.01 | 164.28 | **-2.33** | 0.0408 |
| TC08002253.hg.1 | **EGR3** | 37.79 | 85.04 | **-2.25** | 0.0131 |
| TC0X001608.hg.1 | **SYAP1** | 2272.40 | 5077.84 | **-2.24** | 0.0004 |
| TC17002040.hg.1 | **ATP1B2** | 108.38 | 227.54 | **-2.1** | 0.0467 |
| TC17002437.hg.1 | **MIR22HG** | 530.06 | 1089.92 | **-2.05** | 0.0004 |
| TC0X001624.hg.1 | **SAT1** | 38698.77 | 77397.54 | **-1.99** | 0.009 |
| TC12002493.hg.1 | **DUSP6** | 59.30 | 112.21 | **-1.9** | 0.0392 |
| TC02004331.hg.1 | **ADD2** | 195.36 | 359.54 | **-1.84** | 0.0194 |
| TC08002252.hg.1 | **LOC286058** | 37.79 | 67.18 | **-1.78** | 0.0019 |
| TC07002236.hg.1 | **AQP1** | 68.59 | 118.60 | **-1.73** | 0.0229 |
| TC10002105.hg.1 | **DDX21** | 6338.83 | 11036.54 | **-1.73** | 0.0387 |
| TC05003160.hg.1 | **CHD1** | 16384.00 | 27554.49 | **-1.68** | 0.0075 |
| TC08002257.hg.1 | **TNFRSF10B** | 604.67 | 996.00 | **-1.64** | 0.0481 |

| ProbeID | Gene Symbol | CHORIO Group Average Expression | Control Group Average Expression | Fold Change | P-value |
|---|---|---|---|---|---|
| TC08002367.hg.1 | **PTTG3P** | 53.82 | 86.82 | **-1.61** | 0.0219 |
| TC01004453.hg.1 | **IL23R** | 45.25 | 69.55 | **-1.53** | 0.032 |
| TC09001882.hg.1 | **UBAP1** | 4544.80 | 6936.54 | **-1.52** | 0.0238 |
| TC17002039.hg.1 | **ATP1B2** | 82.71 | 124.50 | **-1.5** | 0.0204 |
| TC0X001292.hg.1 | | 233.94 | 643.59 | **-2.76** | 0.0152 |
| TC10001182.hg.1 | | 9410.14 | 20738.16 | **-2.2** | 0.0137 |
| TC12000140.hg.1 | | 568.10 | 1226.22 | **-2.16** | 0.0493 |
| TC03000057.hg.1 | | 461.44 | 867.07 | **-1.89** | 0.0313 |
| TC02000821.hg.1 | | 11.63 | 20.97 | **-1.8** | 0.018 |
| TC02000833.hg.1 | | 11.63 | 20.97 | **-1.8** | 0.018 |
| TC02000835.hg.1 | | 11.63 | 20.97 | **-1.8** | 0.018 |
| TC02002302.hg.1 | | 11.63 | 20.97 | **-1.8** | 0.018 |
| TC02002305.hg.1 | | 11.63 | 20.97 | **-1.8** | 0.018 |
| TC02002315.hg.1 | | 11.63 | 20.97 | **-1.8** | 0.018 |
| TC02002316.hg.1 | | 11.63 | 20.97 | **-1.8** | 0.018 |
| TC02002337.hg.1 | | 11.63 | 20.97 | **-1.8** | 0.018 |
| TC14000014.hg.1 | | 11.63 | 20.97 | **-1.8** | 0.018 |
| TC14000020.hg.1 | | 11.63 | 20.97 | **-1.8** | 0.018 |
| TC14000024.hg.1 | | 11.63 | 20.97 | **-1.8** | 0.018 |
| TC14000860.hg.1 | | 11.63 | 20.97 | **-1.8** | 0.018 |
| TC14000863.hg.1 | | 11.63 | 20.97 | **-1.8** | 0.018 |
| TC14000866.hg.1 | | 11.63 | 20.97 | **-1.8** | 0.018 |
| TC15001030.hg.1 | | 11.63 | 20.97 | **-1.8** | 0.018 |
| TC18000390.hg.1 | | 11.63 | 20.97 | **-1.8** | 0.018 |
| TC21000020.hg.1 | | 11.63 | 20.97 | **-1.8** | 0.018 |
| TC22000442.hg.1 | | 11.63 | 20.97 | **-1.8** | 0.018 |
| TC22000445.hg.1 | | 11.63 | 20.97 | **-1.8** | 0.018 |
| TC22000450.hg.1 | | 11.63 | 20.97 | **-1.8** | 0.018 |
| TC10000551.hg.1 | | 210.84 | 367.09 | **-1.74** | 0.005 |
| TC15001956.hg.1 | | 24.25 | 41.93 | **-1.72** | 0.044 |
| TC06000878.hg.1 | | 28.25 | 48.50 | **-1.71** | 0.0492 |
| TC06001629.hg.1 | | 29.45 | 50.21 | **-1.7** | 0.0203 |
| TC11000068.hg.1 | | 39.12 | 65.80 | **-1.68** | 0.0028 |
| TC06001641.hg.1 | | 14.72 | 24.76 | **-1.67** | 0.0087 |

| ProbeID | Gene Symbol | CHORIO Group Average Expression | Control Group Average Expression | Fold Change | P-value |
|---|---|---|---|---|---|
| TC10000827.hg.1 | | 138.14 | 229.13 | **-1.67** | 0.0385 |
| TC0Y000099.hg.1 | | 19.70 | 32.00 | **-1.62** | 0.0472 |
| TC0Y000204.hg.1 | | 19.70 | 32.00 | **-1.62** | 0.0472 |
| TC11000813.hg.1 | | 160.90 | 259.57 | **-1.61** | 0.0417 |
| TC10000130.hg.1 | | 191.34 | 306.55 | **-1.6** | 0.0054 |
| TC11001759.hg.1 | | 179.77 | 284.05 | **-1.57** | 0.0358 |
| TC14000432.hg.1 | | 621.67 | 975.50 | **-1.57** | 0.0321 |
| TC06001181.hg.1 | | 20.68 | 32.00 | **-1.55** | 0.0005 |
| TC15000546.hg.1 | | 18.38 | 28.44 | **-1.54** | 0.0149 |
| TC07000685.hg.1 | | 37.27 | 56.89 | **-1.53** | 0.0341 |
| TC17001909.hg.1 | | 86.22 | 131.60 | **-1.52** | 0.0433 |
| TC14000637.hg.1 | | 74.03 | 112.21 | **-1.52** | 0.0492 |
| TC19000183.hg.1 | | 3169.41 | 4770.75 | **-1.51** | 0.0478 |
| TC06001872.hg.1 | | 129.79 | 195.36 | **-1.51** | 0.0275 |
| TC05001712.hg.1 | | 12.38 | 18.64 | **-1.51** | 0.0069 |
| TC01002961.hg.1 | | 229.13 | 344.89 | **-1.5** | 0.0322 |
| TC03003282.hg.1 | | 393.44 | 2241.11 | **-5.7** | 0.0038 |
| TC05003141.hg.1 | | 218.27 | 634.73 | **-2.91** | 0.0051 |
| TC11002782.hg.1 | | 108.38 | 225.97 | **-2.09** | 0.0112 |
| TC20001155.hg.1 | | 43.41 | 88.03 | **-2.03** | 0.0185 |
| TC13001355.hg.1 | | 45702.96 | 85877.94 | **-1.87** | 0.023 |
| TC06003961.hg.1 | | 14.62 | 26.91 | **-1.85** | 0.0371 |
| TC09001960.hg.1 | | 27.10 | 48.84 | **-1.8** | 0.0057 |
| TC0X001908.hg.1 | | 32995.92 | 58656.36 | **-1.78** | 0.0301 |
| TC14002072.hg.1 | | 71.01 | 122.79 | **-1.73** | 0.0078 |
| TC13000992.hg.1 | | 24.25 | 39.67 | **-1.64** | 0.0006 |
| TC21000824.hg.1 | | 19.70 | 31.34 | **-1.6** | 0.0069 |
| TC01005996.hg.1 | | 533.74 | 849.22 | **-1.59** | 0.0245 |
| TC09002600.hg.1 | | 45.89 | 73.01 | **-1.59** | 0.01 |
| TC05002657.hg.1 | | 64.00 | 100.43 | **-1.57** | 0.034 |
| TC15002185.hg.1 | | 49.87 | 77.71 | **-1.56** | 0.0208 |
| TC09001920.hg.1 | | 56.49 | 87.43 | **-1.55** | 0.0274 |
| TC21000612.hg.1 | | 23.10 | 35.51 | **-1.53** | 0.0075 |
